# 

**Published:** 1888-03

**Authors:** 


CONTENTS:

Article I.—The Pathology of Alveolar Abscess. By E. Lloyd Williams, M. R. C. S., L. R. C. P., L. D. S... 481

" II.—A Very Valuable Lesson for Those Who Use Anæsthetics. By Prof. Julian J. Chisolm, M. D. .......... 494

" III.—A Misplaced Right Superior Cuspid Tooth, With Resulting Sequelæ. By W. W. H. Thackston, M. D., D. D. S .......... 510

" IV.—Ammonia vs. Zinc Fillings. By Levitt E. Custer, B. S., D. D. S .......... 515

" V.—On the Causes of Acute Suppurations in the Light of Present Scientific Views. By A. Luckerman.......... 517

MONTHLY SUMMARY.

The Application of Herbst's Method to Tin Gold Fillings.......... 519

Dark Joints.......... 520

Perseverance an Important Factor.......... 521

Thorough Examination.......... 522

A Voice Raised Against Carpets.......... 522

Experiments.......... 523

What is Pain?.......... 523

Gold is and Will Remain the King of Filling Materials.......... 524

Flies in the Operating Room.......... 524

Anchor Pins.......... 525

The Cultivation of the Faculty of Knowing.......... 525

Cocaine and Carbolic Acid Anæsthesia.......... 526

Talent vs. Genius.......... 526

Poisoning With Iodol.......... 527

In Regard to Obtundents.......... 528

Against Indiscriminate Use of Tea and Coffee.......... 528

Lubricating Jack Screws.......... 528

